# Sex- and age-specific clinical and immunological features of coronavirus disease 2019

**DOI:** 10.1371/journal.ppat.1009420

**Published:** 2021-03-26

**Authors:** Shengwei Jin, Hui An, Tong Zhou, Ting Li, Mengzhen Xie, Saijing Chen, Chengshui Chen, Binyu Ying, Zhangye Xu, Xiaokun Li, Ming Li

**Affiliations:** 1 School of Basic Medical Science, Wenzhou Medical University, Wenzhou, China; 2 Department of Anesthesia and Critical Care, The Second Affiliated Hospital and Yuying Children’s Hospital of Wenzhou Medical University, Zhejiang, China; 3 Department of Gynecology and Obstetrics, The Second Affiliated Hospital of Wenzhou Medical University, Wenzhou, China; 4 Department of Pulmonary and Critical Care Medicine, The First Affiliated Hospital of Wenzhou Medical University; Wenzhou, China; 5 Department of Critical Care Medicine, The Second Affiliated Hospital and Yuying Children’s Hospital of Wenzhou Medical University, Wenzhou, Zhejiang, China; 6 School of Pharmacy, Wenzhou Medical University, Wenzhou, Zhejiang, China; Johns Hopkins University Bloomberg School of Public Health, UNITED STATES

## Abstract

To simultaneously determine clinical and immunological responses to severe acute respiratory syndrome coronavirus 2 (SARS-CoV-2) infection in young and old females and males, 681 coronavirus disease 2019 (COVID-19) patients and 369 normal controls (NCs) were analyzed based on age and sex classifications using multiple linear regression analysis. Compared to the age-matched NCs, both young and old male and female non-comorbid COVID-19 patients had lower lymphocyte counts and alanine aminotransferase (ALT) concentration, and only young male and female patients had lower neutrophil counts. Compared to young patients, both old males and females had significantly higher plasma ALT and AST concentrations. Compared to young and old females, age-matched males had higher plasma ALT and AST concentrations, but only young males had higher C-reactive protein (CRP) concentration. Compared to females, old males, but not young males, showed higher incidence of critical illness. Compared to young patients, old females had more leukocyte and neutrophil counts above the normal upper limit and B cell count below the normal lower limit (NLL), while old males had more lymphocyte and natural killer (NK) cell counts below the NLL. No sex or age associations with B cell and NK cell counts were observed. However, there were age-dependent decreases in CD8^+^ T-cell counts in both male and female COVID-19 patients. Age was negatively associated with CD8^+^ T cell counts but positively associated with neutrophil count, CRP, ALT, and AST concentrations, and sex (females) was negatively associated with neutrophil count, CRP, ALT, and AST concentrations. The present study suggests that SARS-CoV-2 infection mainly induced 1) beneficial sex (female)-related differences regarding reduced COVID-19 disease severity and negative associations with inflammatory responses and liver damage, and 2) harmful age-related differences relating to negative associations with CD8^+^ T cell count and positive associations with inflammatory responses and liver damage. Thus, sex and age are biological variables that should be considered in the prevention and treatment of COVID-19.

## Author summary

Previous studies to estimate sex- or age-specific differences in coronavirus disease 2019 (COVID-19) have not considered the differences in immune profiles and disease susceptibility patterns across life, attributed to variations in age and sex, and resulting in sex-age-confluent results. In this retrospective study, we assigned adult COVID-19 patients into four groups according to sex and age and simultaneously determined the features of sex- and age-specific clinical and immunological responses to SARS-CoV-2 infection. SARS-CoV-2 infection mainly induced 1) beneficial sex (female)-related differences regarding reduced COVID-19 disease severity and negative associations with inflammatory responses and liver damage, and 2) harmful age-related differences regarding negative associations with CD8^+^ T cell numbers and positive associations with inflammatory responses and liver damage. The present findings emphasize that COVID-19 pathogenesis and treatment should be tailored according to sex and age.

## Introduction

Sex-disaggregated data from the ongoing coronavirus disease 2019 (COVID-19) pandemic indicates that severe acute respiratory syndrome coronavirus 2 (SARS-CoV-2) infection causes disproportionate mortality in males. Reports from 37 of the 38 countries that have provided sex-disaggregated data demonstrate that COVID-19 fatality rates are higher in males than in females [1–4]. Scully et al. showed that the average male case fatality rate (CFR) across 38 countries is 1.7 times higher than the average female CFR [4]. The current understanding of sex- and age-dependent differences in COVID-19 is very limited. Most published studies of sex differences utilize all patients with COVID-19 and do not address whether sex differences in clinical and immune function change with aging. An early analysis based on 168 patients with severe COVID-19 demonstrated differences in male and female laboratory parameters, including neutrophil/lymphocyte ratios (NLR), C-reactive protein (CRP), ferritin, alanine aminotransferase (ALT), aspartate aminotransferase (AST), blood urea nitrogen, and creatinine levels [5]. In explaining the sexual dimorphism in the clinical and immunological features of COVID-19, both biological sex and age factors should be addressed simultaneously. This is because the differences in immune profiles and disease susceptibility patterns throughout life are attributable to variation in sex steroid concentrations, which affects both innate and adaptive immune responses at different ages [6,7]. Several data pieces have shown that the effects of sex and age on infection pathogenesis are intertwined [8,9]. However, simultaneous studies on sex- and age-specific differences in clinical and immune characteristics in COVID-19 patients are scarce. Thus, in this study, sex- and age-specific differences in the clinical and immunological characteristics of SARS-CoV-2 infection were simultaneously highlighted.

## Results

### Baseline characteristics of male and female patients with COVID-19

From January 17, 2020, to March 20, 2020, a total of 693 patients were identified with laboratory-confirmed SARS-CoV-2 infection in 10 hospitals in Wenzhou, China. Of these patients, only 12 were below 17 years old; thus, 681 met the inclusion criteria for the present analysis (S1 Table). The median patient age in 362 males (53.2%) was 47.5 years (interquartile range [IQR] 39–55) and 48.0 years (IQR 37.5–56.0) in 319 females (46.8%). In the young cohort, the COVID-19 incidence was significantly higher in males (54.0%) than in females (46.0%, *p* < 0.05). A total of 243 (35.7%) of 681 patients had comorbidities, with hypertension being the most common, followed by diabetes, coronary heart disease, chronic liver disease, chronic kidney disease, cancers, immune disorders, and carcinoma. In the young patients, more male patients had comorbidities (25.2%) than did female patients (15.7%, *p* < 0.05, S1 Table). Removing confounders, such as the prevalence of comorbidities, in study cohorts improves the analysis of specific effects of COVID-19. Of 681 patients, 438 (223 males and 215 females) suffered from COVID-19 without comorbidities. Of these 438 patients, 285 were young (65.0%, 146 males and 139 females) and 153 were old (35.0%, 77 males and 76 females). Compared to old females, old males experienced more critical illness (none *vs*. 9.1%, *p* < 0.01). Compared to young patients, old females but not old males had more severe cases (5.0% *vs*. 19.1%, *p* < 0.01). The most common clinical manifestations in patients with COVID-19 at the onset of illness included fever, dry cough, sputum production, and fatigue. Less common symptoms included diarrhea, nausea or vomiting, headache, myalgia or arthralgia, and coeliodynia. Among all signs and symptoms, there was a significant difference in diarrhea between young and old males (19.2% *vs*. 6.5%, *p* < 0.05). In female patients, old patients more frequently had an oxygen index < 300 mmHg (*p* < 0.05) compared to young patients (5.0% *vs*. 19.1%, *p* < 0.01).

### Laboratory findings of male and female patients with COVID-19

In this study, the increase or decrease in cell counts and biochemical parameters were defined as above or under the limit of normal ranges according to local hospital criteria. Compared to the young and old normal controls (NCs), both age-matched male and female COVID-19 patients had more cases with leukocyte and lymphocyte counts below the normal lower limit (NLL) and ALT concentrations above the normal upper limit (NUL). Compared to young males with COVID-19, the age-matched female patients had more cases with leukocyte counts, plasma ALT, and urea nitrogen concentrations below the NLL (Table 1). Compared to young patients, old females had more cases with leukocyte and neutrophil counts above the NUL, and old males had more cases with lymphocyte counts below the NLL (Table 1). These data resulted in a markedly higher neutrophil-to-lymphocyte ratio (NLR) in both old sexes than in young cohorts (both *p* < 0.05). No difference in the NLR between sexes was observed. Compared to normal ranges, SARS-Cov-2 infection was associated with lymphocyte subset cell counts below the NLL in males and females, respectively, as follows: CD4^+^ T cells: 55.9% and 38.4%; CD8^+^ T cells: 56.9% and 45.3%; B cells: 79.0% and 79.6%; and NK cells: 30.2% and 19.6%. Compared to young patients, old females had fewer cases with B cell counts below the NLL (91.7% vs. 64.3%, *p* < 0.05), and old males had more cases with NK cell counts below the NLL (19.4% vs. 44.4%, *p*<0.05, Table 1). No difference in CD8^+^ T cell count was observed between the sexes. However, there were age-dependent decreases in CD8^+^ T cell count in both male (young cohort 332 cells/μL [IQR 239–502] vs. old cohort 216 cell/μL [IQR 133–321], *p* < 0.001) and female (young cohort 374 cells/μL [IQR 285–493] vs. old cohort 226 cell/μL [IQR 143–392], *p* < 0.01) COVID-19 patients.

**Table 1:** Sex- and Age-specific Clinical and Laboratory Characteristics of Normal Controls and COVID-19 Patients.

	Reference ranges	Normal controls				Covid-19 Patients				P-value^e^	P-value^f^	P-value^g^	P-value^h^	P-value^i^	P-value^j^	P-value^k^	P-value^l^
		Yong (n = 263)		Old (n = 106)		Yong (n = 285)		Old (n = 153)									
		Males (n = 160)	Females (n = 103)	Males (n = 50)	Females (n = 56)	Males (n = 146)	Females (n = 139)	Males (n = 77)	Females (n = 76)								
Age, years		26 (23–33)	25 (22–32)	57(54–64)	56(53–63)	39 (31–43)	38 (32–44)	55 (53–59)	56 (53–64)	-	-	-	-	-	-	-	-
Sex		160/263 (60.8)	103/263 (39.2)	50/106 (47.2)	56/106 (52.8)	146/285 (51.2)	139/285 (48.8)	77/153 (50.3)	76/153 (49.7)	-	-	-	-	-	-	-	-
**Severity**																	
Mild		-	-	-	-	101/146 (69.2)	95/139 (68.3)	57/77 (74.0)	55/76 (72.4)	-	-	0.449	0.539	-	-	-	-
Moderate		-	-	-	-	1/146 (0.7)	0/139 (0)	1/77 (1.3)	0/76 (0)	-	-	>0.999	>0.999	-	-	-	-
Severe		-	-	-	-	13/115 (11.3)	5/100 (5.0)	8/66 (12.1)	13/68 (19.1)		-	0.869	0.004	-	-	-	-
Critical ill		-	-	-	-	1/146 (0.7)	1/139 (0.7)	7/77 (9.1)	0/76 (0)d	-		0.005	>0.999	-	-	-	-
**Clinical symptoms**																	
Fever		-	-	-	-	97/146 (66.4)	86/139 (61.9)	48/77 (62.3)	51/76 (67.1)	-	-	0.542	0.445	-	-	-	-
Dry cough		-	-	-	-	63/146 (43.2)	48/139 (34.5)	30/77 (39.0)	26/76 (34.2)	-	-	0.546	0.962	-	-	-	-
Sputum production		-	-	-	-	76/146 (52.1)	63/139 (45.3)	43/77 (55.8)	42/76 (55.3)	-	-	0.590	0.163	-	-	-	-
Fatigue		-	-	-	-	38/146 (26.0)	41/139 (29.5)	22/77 (28.6)	25/76 (32.9)	-	-	0.684	0.606	-	-	-	-
Myalgia		-	-	-	-	10/146 (6.8)	11/139 (7.9)	6/77 (7.8)	5/76 (6.6)	-	-	0.795	0.722	-	-	-	-
Headache		-	-	-	-	15/146 (10.3)	12/139 (8.6)	11/77 (14.3)	10/76 (13.2)	-	-	0.375	0.295	-	-	-	-
Nausea or vomiting		-	-	-	-	13/146 (8.9)	11/139 (7.9)	2/77 (2.6)	8/76 (10.5)	-	-	0.132	0.519	-	-	-	-
Coeliodynia		-	-	-	-	3/146 (2.1)	7/139 (5.0)	1/77 (1.3)	3/76 (3.9)	-	-	>0.999	0.981	-	-	-	-
Diarrhea		-	-	-	-	28/146 (19.2)	28/139 (20.1)	5/77 (6.5)	8/76 (10.5)	-	-	0.011	0.071	-	-	-	-
Oxygen index, mmHg	>300	-	-	-	-	428(367–474)	448(382–500)	400 (338–460)	400 (327–466)	-	-	>0.999	0.031	-	-	-	-
<300 mmHg		-	-	-	-	13/115 (11.3)	5/100 (5.0)	8/66 (12.1)	13/68 (19.1)	-	-	0.869	0.004	-	-	-	-
**Laboratory parameters**																	
Leukocyte count, ×10^9^ /L	4–10	6.0 (5.3–7.0)	5.7 (4.9–6.9)	5.8(5.2–6.3)	5.4(4.7–6.5)	5.0 (3.8–6.4)	4.3 (3.4–5.4)^c^	5.5 (4.4–7.4)	4.6 (3.9–6.4)	>0.999	>0.999	0.884	0.371	<0.0001	>0.999	<0.0001	>0.999
<4 ×10^9^ /L		7/160 (4.4)	2/103 (2.0)	2/49(4.1)	4/55(7.1)	39/143 (27.3)	51/138 (37.0)	14/77 (18.2)	21/76 (27.6)	>0.999	0.217	0.133	0.167	<0.0001	0.020	<0.0001	0.003
>10 ×10^9^ /L		1/160 (0.6)	2/103 (1.9)	0/49(0)	0/55(0)	8/143 (5.6)	0/138 (0)^c^	5/77 (6.5)	8/76 (10.5)	>0.999	>0.999	0.787	0.0002	0.027	0.176	0.182	0.035
Neutrophil count, ×10^9^ /L	1.8–6.3	3.5 (2.9–4.4)	3.5 (2.9–4.4)	3.4(2.7–3.9)	3.3(2.4–4.1)	3 (2.1–4.1)	2.7 (1.8–3.8)	3.4 (2.6–4.7)	3.1 (2.2–4.6)	>0.999	>0.999	0.607	0.172	0.009	>0.9999	<0.0001	>0.9999
<1·8 ×10^9^ /L		1/160 (0.6)	0/103 (0)	0/49(0)	2/55(3.6)	21/142 (14.8)	33/138 (23.9)	11/77 (14.3)	9/76 (11.8)	>0.999	0.230	0.920	0.033	<0.0001	0.014	<0.0001	0.176
>6·3 ×10^9^ /L		6/160 (3.8)	5/103 (4.9)	1/49(2.0)	0/55(0)	10/142 (7.0)	1/138 (0.7)cc	11/77 (14.3)	8/76 (10.5)	>0.999	0.237	0.082	0.002	0.202	0.049	0.409	0.035
Lymphocyte count, ×10^9^ /L	1.1–3.2	1.9 (1.5–2.2)	1.8 (1.5–2.0)	1.9(1.58–2.18)	1.9(1.5–2.3)	1.3 (1.0–1.7)	1.3 (1.0–1.5)	1.2 (0.9–1.5)	1.2 (0.9–1.5)	>0.999	>0.999	>0.999	>0.999	<0.0001	<0.0001	<0.0001	<0.0001
<1.1 ×10^9^ /L		2/160 (1.3)	1/103 (0.97)	2/49(4.1)	1/55(1.8)	39/143 (27.3)	41/138 (29.7)	31/77 (40.3)	30/76 (39.5)	0.503	<0.0001	0.049	0.147	<0.0001	<0.0001	<0.0001	<0.0001
NLR		1.8 (1.4–2.5)	2.0 (1.6–2.4)	1.8(1.4–2.3)	1.7(1.3–2.2)	2.2 (1.5–3.2)	2.0 (1.4–2.9)	2.7 (1.9–4.4)	2.7 (1.8–3.7)	>0.999	0.723	0.300	0.041	0.365	0.0003	>0.999	<0.0001
Hematocrit	M: 40–50 F: 35–45	45.4 (43.6–47.0)	40.0 (37.9–41.3)aaaa	46.4(44.65–48.2)	41.5(39.1–43.9)bbbb	43.6 (41.7–45.8)	38.1 (35.2–40.2)cccc	41.4 (38.4–43.8)	37.1 (34.9–39.7)dddd	>0.999	0.063	0.001	>0.999	0.011	<0.0001	0.938	<0.0001
Male < 40; Female < 35		0/160 (0)	5/103 (4.9)^a^	0/49(0)	1/55(1.82)	20/139 (14.4)	31/137 (22.6)	28/76 (36.8)	19/74 (25.7)	>0.999	0.607	0.0002	0.619	<0.0001	<0.0001	<0.0001	0.001
Hemoglobin, g/L	M:120–160 F:110–150	156.0(149.0–162.0)	133.0(128.0–139.0)aaaa	154.0(150.5–160.0)	136.0(128.0–143.0)bbbb	150.0 (141.8–156.0)	128.0 (122.3–135.0)cccc	139.0 (131.5–148.0)	123.5 (116.0–131.0)dddd	>0.999	>0.999	0.0004	>0.999	0.011	<0.0001	<0.0001	0.003
Fibrinogen, g/L	2–4	-	-	-	-	3.9 (3.1–4.8)	3.3 (2.8–4.3)^c^	4.1 (3.3–4.8)	4.1 (3.3–4.6)	-	-	>0.999	0.003	-	-	-	-
>4 g/L		-	-	-	-	54/128 (42.2)	36/119 (30.3)	39/69 (56.5)	37/70 (52.9)	-	-	0.055	0.002	-	-	-	-
D-dimer, mg/L	<500	-	-	-	-	220 (143–350)	230 (130–345)	280 (160–500)	285 (195–498)	-	-	0.411	0.101	-	-	-	-
>500 mg/L		-	-	-	-	21/124 (16.9)	16/115 (13.9)	16/69 (23.2)	17/70 (24.3)	-	-	0.290	0.074	-	-	-	-
cTn-, ng/L	<0.01	-	-	-	-	0.012 (0.005–0.014)	0.01 (0.001–0.012)c	0.01 (0.006–0.02)	0.011 (0.008–0.013)	-	-	>0.999	0.097	-	-	-	-
>0.01 ng/L		-	-	-	-	55/99 (55.6)	40/92 (43.5)	28/57 (49.1)	28/55 (50.9)	-	-	0.438	0.382	-	-	-	-
BNP, pg/mL	<100	-	-	-	-	14 (10–100)	32 (10–100)	44(10–100)	35 (10–100)	-	-	>0.999	>0.999	-	-	-	-
Albumin, g/L	40–55	46.9 (45.5–48.6)	45.5 (43.9–46.7)aa	46.8(45.5–47.9)	46.7(45.5–47.9)	42.1 (39.2–44.8)	41.4 (38.8–43.6)	38.6 (35.7–41.0)	39.2 (36.2–41.6)	>0.999	0.169	0.002	0.152	<0.0001	<0.0001	<0.0001	<0.0001
<40		0/162(0)	1/103(0.97)	0/50(0)	0/56(0)	45/141(31.9)	51/131(38.9)	48/74(64.9)	45/76(59.2)	>0.999	>0.999	<0.0001	<0.0001	<0.0001	<0.0001	<0.0001	<0.0001
Globulin, g/L	20–30	-	-	23.9(22.5–26.1)	25.3(24.1–27.7)	27.5 (24.4–30.8)	28.4 (25.3–30.3)	28.8 (26.2–32.1)	29.1 (26.2–33.3)	-	-	0.054	0.646	-	<0.0001	-	<0.0001
>30		-	-	1/50(2.0)	4/56(7.1)	42/141(29.8)	38/131(29.0)	27/74(36.5)	31/76(40.8)	-	-	0.227	0.083	-	<0.0001	-	<0.0001
ALT, IU/L	M: 5–40 F: 5–35	17.0 (13.0–25.0)	11.0 (9.0–14.0)aaaa	21.0(17.8–26.0)	17.0(14.0–21.8)	27.0 (17.1–42.0)	13.0 (11.0–21.0)cccc	29.0 (21.0–50.5)	18.8 (14.0–27.5)dd	>0.999	<0.0001	>0.999	0.011	<0.0001	0.308	0.0008	>0.999
Male >40 IU/L; Female >35 IU/L		12/160 (7.5)	2/103 (1.9)	2/50(4.0)	2/56(3.6)	37/143 (25.9)	11/137 (8.0)cccc	26/76 (34.2)	12/76 (15.8)dd	0.588	0.923	0.195	0.080	<0.0001	<0.0001	0.039	0.024
AST, IU/L	8–40	19.0 (16.0–22.0)	17.0 (15.0–19.0)	21.5(19.75–25.0)	22.5(18.0–25.0)	23.0 (19.0–30.0)	19.0 (16.7–23.0)ccc	28.0 (23.0–42.8)	26.0 (19.0–31.0)	0.015	<0.0001	0.086	<0.0001	<0.0001	0.030	0.0005	>0.999
>40 IU/L		5/160 (3.1)	1/103 (0.97)	0/50(0)	0/56(0)	15/129 (11.6)	7/129 (5.4)	19/71 (26.8)	12/73 (16.4)	0.463	>0.999	0.006	0.01	0.005	<0.0001	0.137	0.001
Total bilirubin, μmol/L	3.4–17.1	15.1 (12.1–19.5)	11.7 (9.4–14.0)aaaa	13.8(11.7–19.0)	12.4(9.9–14.4)	11.5 (8.8–15.3)	9.0 (6.4–13.5)c	11.3 (8.1–14.1)	10.1 (6.3–16.3)	>0.999	>0.999	>0.999	>0.9999	<0.0001	0.043	0.0205	>0.999
Direct Bilirubin, μmol/L	0–3.4	4.0 (3.2–5.5)	3.0 (2.4–3.9)aaaa	3.1(2.6–4.2)	2.6(2.0–3.3)	4.1 (3.0–5.6)	3.3 (2.5–4.9)cc	3.9 (3.0–5.9)	3.6 (2.7–5.9)	0.027	0.645	>0.999	>0.9999	>0.9999	0.347	>0.9999	<0.0001
Urea nitrogen, mmol/L	M:2.3–7.1; F: 1.8–6.1	4.7 (3.9–5.3)	3.9 (3.3–4.6)aa	6.0(5.1–6.9)	5.3(4.5–6.1)	3.9 (3.4–4.5)	3.1 (2.7–3.5)cccc	4.3 (3.6–5.3)	3.7 (2.9–4.7)d	<0.0001	<0.0001	0.072	0.002	<0.0001	<0.0001	<0.0001	<0.0001
Male > 7.1 mmol/L; Female > 6.1mmol/L		6/160 (3.8)	3/99 (3.0)	10/50(20.0)	13/55(23.6)	1/143 (0.7)	0/137 (0)	5/76 (6.6)	6/76 (7.9)	0.001	<0.0001	0.036	0.002	0.167	0.023	0.144	0.012
Creatinine, μmol/L	M:62–115; F: 53–97	69.6 (64.3–74.8)	50.8 (46.2–56.1)aaaa	71.3(66.4–76.2)	51.8(46.6–58.1)bbbb	76.0 (67.0–86.5)	56.9 (50.9–62.0)cccc	71.8 (63.9–84.0)	58.0 (51.0–65.0)dddd	>0.999	>0.999	>0.999	>0.999	0.022	>0.999	0.003	0.166
Male > 115 μmol/L; Female > 97 μmol/L		0/160 (0)	0/99 (0)	0/50(0)	0/55(0)	0/143 (0)	0/138 (0)	1/76 (1.3)	0/76 (0)	>0.999	>0.999	0.347	>0.999	>0.999	>0.999	>0.999	>0.999
**Inflammatory factors- cytokines and interleukins**																	
CRP, mg/L	≤10	-	-	0.95(0.44–1.30)	0.73(0.50–1.28)	8.8 (4.7–22.0)	5.0 (2.2–13.7)cc	12.8 (5.0–36.9)	11.0 (5.0–22.1)	-	-	>0.999	0.027	-	<0.0001	-	<0.0001
≥10 pg/mL		-	-	0/30(0)	2/35(5.7)	67/141 (47.5)	40/136 (29.4)cc	44/76 (57.9)	39/76 (51.3)	-	-	0.145	0.002	-	<0.0001	-	<0.0001
IFN-γ, pg/mL	2.55–4.37	-	-	-	-	2.5 (1.4–3.3)	2.8 (1.7–3.5)	2.2 (0.8–2.8)	1.9 (1.0–2.8)	-	-	>0.999	0.325	-	-	-	-
>4.4 pg/mL		-	-	-	-	8/55 (14.5)	8/45 (17.8)	3/27 (11.1)	5/38 (13.2)	-	-	0.933	0.564	-	-	-	-
TNF-α, pg/mL	1.05–1.95	-	-	-	-	1.4 (0.8–2.5)	1.1 (1.0–2.1)	0.8 (0.5–2.1)	1.0 (0.5–1.7)	-	-	0.469	>0.999	-	-	-	-
>2 pg/mL		-	-	-	-	19/55 (34.5)	12/45 (26.7)	9/27 (33.3)	8/38 (21.1)	-	-	0.913	0.551	-	-	-	-
IL-2, pg/mL	1.07–1.43	-	-	-	-	1.5 (1.1–2.5)	1.6 (1.3–2.5)	1.1 (0.6–2.5)	1.5 (1.1–2.2)	-	-	0.814	>0.999	-	-	-	-
>1.5 pg/mL		-	-	-	-	27/55 (49.1)	23/45 (51.1)	11/27 (40.7)	14/38 (36.8)	-	-	0.476	0.193	-	-	-	-
IL-4, pg/mL	1.13–1.71	-	-	-	-	1.9 (1.2–2.8)	1.8 (1.2–2.5)	1.3 (0.6–2.5)	1.5 (1.1–2.0)	-	-	0.640	>0.999	-	-	-	-
>1.8 pg/mL		-	-	-	-	28/55 (50.9)	21/45 (46.7)	11/27 (40.7)	12/38 (31.6)	-	-	0.386	0.162	-	-	-	-
IL-5, pg/ml		-	-	-	-	1.6(1.3–2.0)	1.2(1.0–1.5)	2.5(2.4–2.5)	1.2(1.0–1.3)	-	-	>0.999	>0.999	-	-	-	-
IL-6, pg/mL	2.15–12.75	-	-	-	-	4.0 (2.6–11.9)	3.7 (2.1–5.9)	5.8 (3.8–39.2)	4.2 (2.5–11.5)	-	-	0.259	>0.999	-	-	-	-
>12.8 pg/mL		-	-	-	-	11/55 (20.0)	7/45 (15.6)	11/27 (40.7)	9/38 (23.7)	-	-	0.046	0.350	-	-	-	-
IL-10, pg/mL	1.18–2.16	-	-	-	-	3.3 (1.6–4.1)	2.8 (0.9–3.9)	3.5 (2.0–5.8)	3.3 (0.9–4.9)	-	-	>0.999	>0.999	-	-	-	-
>2.2 pg/ml		-	-	-	-	38/55 (69.1)	26/45 (57.8)	19/27 (70.4)	25/38 (65.8)	-	-	0.906	0.455	-	-	-	-
**Lymphocyte and subsets**																	
Lymphocytes μL	1100–3200	-	-	-	-	1300 (1000–1700)	1300 (1000–1523)	1170 (900–1530)	1150 (900–1525)	-	-	0.238	0.996	-	-	-	-
<1100 cells /μl		-	-	-	-	39/143 (27.3)	41/138 (29.7)	31/77 (40.3)	30/76 (39.5)	-	-	0.049	0.147	-	-	-	-
CD4^+^ T cells /μL	>500	-	-	-	-	467.5 (328.0–641.5)	535.0 (405.0–597.8)	460.0 (316.5–703.0)	541.0 (393.0–751.0)	-	-	>0.999	>0.999	-	-	-	-
<500 cells /μL		-	-	-	-	34/62 (54.8)	17/45 (37.8)	23/40 (57.5)	16/41 (39.0)	-	-	0.792	0.906	-	-	-	-
CD8^+^ T cells /μL	320–1250	-	-	-	-	331.5 (239.0–502.3)	374.0 (284.9–493.0)	216.0 (133.3–321.3)	226.0 (143.0–392.0)	-	-	0.0007	0.007	-	-	-	-
<320 cells		-	-	-	-	28/62 (45.2)	13/45 (28.9)	30/40 (75.0)	26/41 (63.4)	-	-	0.003	0.001	-	-	-	-
CD4^+^ T cell / CD8^+^ T cell	1.5–2.0	-	-	-	-	1.4 (1.0–1.8)	1.3 (1.1–1.6)	2.2 (1.6–2.6)	2.5 (1.8–3.1)	-	-	<0.0001	<0.0001	-	-	-	-
T cells /μL	955–2860	-	-	-	-	836.5 (574.0–1202.8)	958.0 (733.0–1134.0)	678.5 (446.3–995.3)	874.0 (536.0–1143.0)	-	-	0.344	>0.999	-	-	-	-
<950 cells /μL		-	-	-	-	37/62 (59.7)	22/45 (48.9)	29/40 (72.5)	23/41 (56.1)	-	-	0.186	0.504	-	-	-	-
B cells /μL	240–560	-	-	-	-	159.0 (133.5–233.5)	131.5 (119.3–168.3)	166.0 (98.0–215.5)	203.5 (148.8–264.5)	-	-	>0.999	0.058	-	-	-	-
<240 cells /μL		-	-	-	-	27/35 (77.1)	22/24 (91.7)	22/27 (81.5)	18/28 (64.3)	-	-	0.677	0.045	-	-	-	-
NK cells /μL	150–1100	-	-	-	-	222.5 (168.3–370.8)	227.5 (157.0–335.3)	184.0 (91.0–299.5)	226.0 (161.5–272.0)	-	-	0.439	>0.999	-	-	-	-
<150 cells /μL		-	-	-	-	7/36 (19.4)	5/24 (20.8)	12/27 (44.4)	5/27 (18.5)^d^	-	-	0.032	0.835	-	-	-	-

Data are median (IQR), n/N (%). p values were calculated by Kruskal-Wallis test, χ^2^ test, Yates’ continuity corrected chi-square test or Fisher’s exact test, as appropriate. yr, years; NLR, neutrophil to lymphocyte ratio; cTn, cardiac troponin; BNP, Brain natriuretic peptide; ALT, alanine aminotransferase; AST, aspartate aminotransferase; CRP, c-reactive protein; IL, interleukin; IFN-γ, interferon-γ.

*a*, *p* values indicate a significant difference compared with young normal males; *a* < 0.05, *aa* < 0.01, *aaa* < 0.001, *aaaa* < 0.0001.

b, p values indicate a significant difference compared with old normal males; *b* < 0.05, *bb* < 0.01, *bbb* < 0.001, *bbbb* < 0.0001.

*c*, *p* values indicate a significant difference compared with young male patients with Covid-19; *c* < 0.05, *cc* < 0.01, *ccc* < 0.001, *cccc* < 0.0001.

*d*, *p* values indicate a significant difference compared with old male patients with Covid-19; *d* < 0.05, *dd* < 0.01, *ddd* < 0.001, *dddd* < 0.0001.

P-value^e^ for comparison of the young vs. old normal males.

P-value^f^ for comparison of the young vs. old normal females.

P-value^g^ for comparison of the young vs. old male patients with COVID-19.

P-value^h^ for comparison of the young vs. old female patients with COVID-19.

P-value^i^ for comparison of the young normal males vs. young male patients with COVID-19.

P-value^j^ for comparison of the old normal males vs. old male patients with COVID-19.

P-value^k^ for comparison of the young normal females vs. young female patients with COVID-19.

P-value^l^ for comparison of the old normal females vs. old female patients with COVID-19.

**Fig 1:**
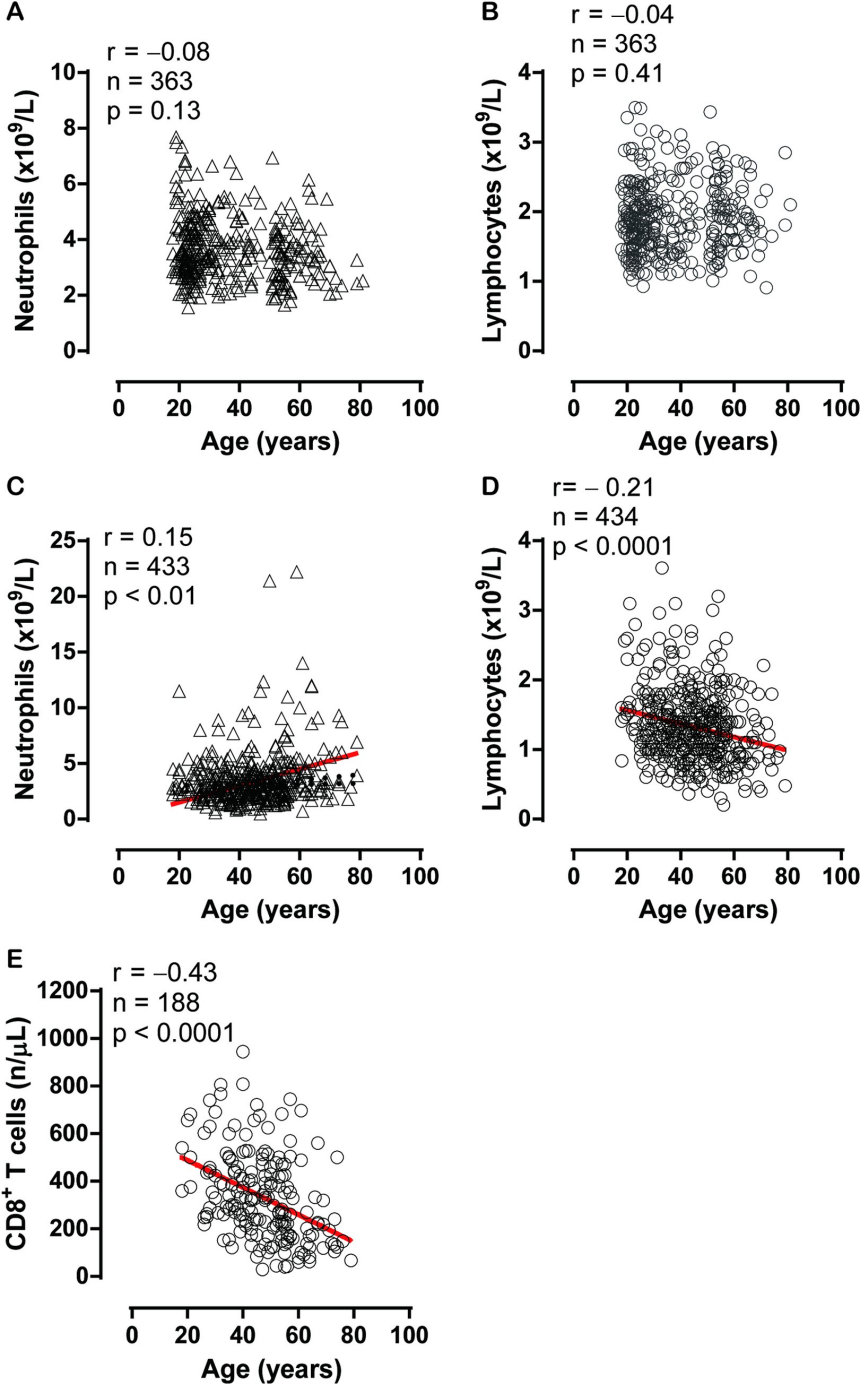
The relationships between neutrophils and age in normal controls (A) (S2 Table) and patients with coronavirus disease 2019 (COVID-19) (C) (S4 Table), lymphocytes and age in normal controls (B) (S3 Table) and patients with COVID-19 (D) (S5 Table), and CD8^+^ T cells and age in patients with COVID-19 (E) (S6 Table).

Spearman correlation analysis revealed that there were significant but weak correlations between age and neutrophil count (Fig 1A, r = 0.15, p < 0.01), as well as between age and lymphocyte count (Fig 1B, r = – 0.21, p < 0.001). A significant and stronger correlation was observed between age and CD8^+^ T cell count (Fig 1C, r = –0.43, p < 0.001).

SARS-Cov-2 infection stimulates inflammatory responses. Compared to the normal ranges, incidences of CRP, interferon (IFN)-γ, tumor necrosis factor (TNF)-α, interleukin (IL)-2, IL-4, IL-6, and IL-10 above the NUL for males with COVID-19 were 51.2%, 13.4%, 34.1%, 46.3%, 47.6%, 26.8%, and 69.5%, and for females with COVID-19, they were 37.3%, 15.7%, 24.1%, 44.6%, 39.8%, 19.3%, and 61.4%, respectively (Table 1). Compared to those in age-matched female cases, young males had significantly higher CRP levels, a marker of tissue damage and inflammation (*p* < 0.01). Plasma fibrinogen (FIB) levels were higher in young males than in age-matched females (*p* < 0.05). Compared to the young cohorts, old females had higher CRP and FIB levels (both *p* < 0.01). In contrast, there were no differences in plasma IFN-γ, TNF-α, IL-2, IL-4, IL-6, and IL-10 levels among sex- and age-matched cohorts with COVID-19. Compared to young patients, both old males and females had significantly higher plasma ALT and AST concentrations. Compared to female patients, age-matched males had higher plasma ALT and AST concentrations, as well as more cases of ALT over the NUL in both young and old cohorts and AST in young patients (Table 1). Furthermore, compared to young patients, old patients of both sexes had lower hypoalbuminemia and higher hyperglobulinemia levels, indicating further age-dependent liver damage in patients with COVID-19.

### Multiple linear regression analysis of innate immune cells, CRP, FIB, ALT and AST levels, and comorbidities

Multiple linear regression analysis (MLRA) revealed that sex (females) and age were negatively and positively associated with neutrophil counts, respectively (β = –0.116 and β = 0.175, p = 0.002 and p = 0.000 respectively). In contrast, no significant associations of sex, age, or comorbidities with lymphocyte, B cell and NK cell counts, and FIB levels were observed (Table 2). Sex, age, and comorbidities were analyzed with MLRA, and the results showed that age was independently negatively associated with CD4^+^ T cell (β = –0.162, p = 0.007) and CD8^+^ T cell counts (β = –0.401, p = 0.000). The MLRA showed that CRP levels were negatively associated with sex (β = –0.133, p = 0.001), but positively associated with age (β = 0.144, p = 0.000), hypertension (β = 0.099, p = 0.03) and diabetes (β = 0.141, p = 0.01). In addition, MLRA revealed that sex was negatively associated with both ALT (β = –0.281, p < 0.000) and AST levels (β = –0.201, p = 0.000), but age was positively associated with ALT (β = 0.114, p = 0.002) and AST levels (β = 0.274, p = 0.000, Table 2).

**Table 2:** Multiple general linear regressions of innate immune cells, c-reactive protein, fibronogen, ALT and AST in patients with COVID-19.

	Sex		Age		Hypertension (HP)		Diabetes (DM)		HP and DM	
	β	p-value	β	p-value	β	p-value	β	p-value	β	p-value
	(95%CI)		(95%CI)		(95%CI)		(95%CI)		(95%CI)	
Neutrophil	-0.116	0.002	0.175	<0.0001	0.022	0.62	0.037	0.50	0.006	0.93
	-1.088 to-0.236		0.021 to 0.052		-0.476 to 0.798		-0.697 to 1.416		-1.474 to 1.619	
Lymphocyte	-0.034	0.38	-0.066	0.09	-0.039	0.38	-0.022	0.69	0.01	0.87
	-0.745 to 0.286		-0.035 to 0.002		-1.092 to 0.415		-1.508 to 1.005		-1.685 to 1.989	
B cell	-0.009	0.91	0.002	0.98	-0.093	0.29	-0.134	0.20	0.071	0.55
	-39.568 to 35.173		-1.245 to 1.276		-85.218 to 25.779		-129.535 to 27.744		-81.485 to 152.266	
NK cell	-0.104	0.16	-0.077	0.30	0.083	0.35	-0.13	0.22	0.142	0.23
	-81.743 to 13.588		-2.462 to 0.752		-37.182 to 105.045		-163.86 to 37.618		-59.063 to 240.333	
CD4^+^ T cell	0.061	0.31	-0.162	0.007	-0.057	0.42	-0.189	0.03	0.036	0.71
	-31.483 to 98.85		-5.42 to-0.858		-140.238 to 58.382		-335.717 to -16.438		-187.101 to 274.724	
CD8^+^ T cell	0.012	0.83	-0.401	<0.0001	-0.016	0.82	-0.132	0.13	0.006	0.95
	-34.699 to 42.971		-6.347 to-3.628		-71.551 to 56.916		-182.279 to 24.23		-144.269 to 154.438	
C-reactive protein	-0.133	0.001	0.144	<0.0001	0.099	0.03	0.141	0.01	-0.074	0.22
	-18.565 to -5.195		0.225 to 0.713		1.423 to 21.045		4.808 to 37.495		-38.597 to 8.927	
Fibronogen	-0.013	0.90	0.011	0.73	0.007	0.88	0.018	0.75	0.044	0.49
	-0.788 to -0.565		-0.021 to 0.028		-0.911 to 1.062		-1.36 to 1.878		-1.534 to 3.202	
ALT	-0.281	<0.0001	0.114	0.002	0.069	0.12	-0.025	0.65	0.003	0.96
	-19.97 to-11.74		0.085 to 0.387		-1.295 to 11.138		-13.006 to 8.132		-14.961 to 15.838	
AST	-0.201	<0.0001	0.274	<0.0001	0.102	0.03	-0.001	0.99	-0.004	0.95
	-12.18 to -5.567		0.321 to 0.564		0.592 to 10.768		-9.121 to 8.947		-13.116 to 12.322	

β, nonstandardized coefficient; CI, confidence interval. ALT = alanine aminotransferase. AST = aspartate aminotransferase.

## Discussion

COVID-19 pathogenesis is based on a potent immunological response involving a complex group of innate immune cells such as neutrophils and lymphocytes. In this retrospective study, we found that SARS-CoV-2 infection decreases blood neutrophil and lymphocyte counts in both male and female patients and damages liver function. Notably, we found beneficial sex (female)-related differences regarding reduced COVID-19 disease severity and negative associations with inflammatory responses and liver damage. We also found harmful age-related differences regarding negative associations with CD8^+^ T cell numbers and positive associations with inflammatory responses and liver damage. Compared to young males, there were more neutropenia and fewer neutrophilia cases in young females with COVID-19, probably due to greater immunologic responses caused by male sex steroids but not by female sex steroids. In healthy young subjects, spontaneous neutrophil apoptosis is significantly decreased in women compared to men. When physiologic doses of estradiol and progesterone were administrated to both men and women, a delay in spontaneous neutrophil apoptosis occurred in both sexes [10]. On the other hand, the androgen receptor (AR) is broadly expressed in neutrophil-lineage cells from the myeloblast stage to the mature neutrophil stage. The androgen stimulates proliferation and differentiation of committed granulocytic precursors and increases neutrophil production [11,12]. Thus, higher neutrophil counts in young males than in females might reflect that young men were exposed to higher androgen levels in the present study. It is well known that the male and female immune systems differ in significant ways, especially after puberty. Particularly, females experience sex-protective responses, such as lower rates of infections and chronic inflammatory diseases. Biologically, genes encoded on sex chromosomes, as well as sex hormones, likely contribute to these differences [8].

In previous studies, data from age- and sex-confluent studies demonstrated that males may be more prone to SARS-CoV-2 infection than females and have a higher risk of poor outcome and fatality [5,13–18]. In Middle East respiratory syndrome coronavirus and SARS-CoV, the same trend has also been observed [19,20]. As shown in Table 1, patients of both sexes without comorbidities showed similar COVID-19 incidences in this study. However, males experienced more critical illness, liver damage (as indicated by ALT and AST levels), higher neutrophil counts, and higher CRP levels than did females. Consistent with previous reports, the present study showed a male predominance in COVID-19-related critical illness incidence, indicating that the more severe illnesses and liver damage in males compared to females were sex-specific effects. This may be partly due to the high expression of angiotensin-converting enzyme-2 (ACE-2), the cell-binding receptor of SARS-CoV-2, which mediates virus entrance in the lungs of men [21], and that 17β-estradiol downregulates lung *ACE2* mRNA and protects females from viral pathogenesis by suppressing inflammatory responses [22]. In terms of laboratory findings, leukocytosis (≥ 10 × 10^9^/L) and neutrophilia (≥ 6.3 × 10^9^/L) were more common in young males than in young females. The higher NLR in males than in females was because of neutrophilia in males, as lymphocyte counts did not differ between sexes in the young cohort. The inflammatory marker, CRP, was significantly higher in young males than in young females, suggesting that, at least partly, inflammatory responses might be associated with sex-specific effects of COVID-19. It is well known that sex-specific disease outcomes may be explained by sex steroids and their activities on X-linked genes and that males and females differ in both innate and adaptive immune responses. The sex-unique mode of inheritance of the X chromosome is attributed to the differences in sex-specific inflammatory responses [23,24].

Previously, age- and sex-confluent results have shown that severe SARS-CoV-2 infection decreases CD4^+^ T cells, CD8^+^ T cells, and NK cells [25,26]. Regarding the age-specific effects of COVID-19, in this study, older patients had lower lymphocyte and CD8^+^ T cell counts, resulting in a higher CD4^+^/CD8^+^ T cell ratio in both sexes. The mechanism underlying significant age-dependent lymphopenia in patients with COVID-19 is unknown and is possibly attributable to aging-related immune senescence [27]. Both human and animal studies have shown that CD8^+^ cytotoxic T cells are critical in mediating viral clearance in human respiratory syncytial virus and influenza A virus infections [28–32]. Thus, cytotoxic immunity (particularly CD8^+^ T cells) might be the key player in determining the age-dependent antiviral processes in patients with COVID-19. Regarding differences in innate immune cells between sexes, interestingly, post-menopausal males had more cases with NK cells below the NLL when compared to post-menopausal females. Another parameter associated with sex-and age-dependent characteristics was the coagulatory marker, FIB, again suggesting that the activation of coagulation/fibrinolysis might be associated with an age-dependent reduction in CD8^+^ T cells. This might reflect the hypothesis that CD8^+^ T cells effectively cleared virus-infected endothelial cells in younger patients but failed to do so in older patients. ACE-2 is the critical receptor on cell membranes for mediating SARS-CoV-2 entry into host cells [33]. Endothelial cells and smooth muscle cells are rich in ACE-2 receptors [34], indicating that these cells may be the targets of virus assault. Therefore, SARS-CoV-2-induced vasculitis might be one of the factors leading to overt disseminated intravascular coagulation, which has been shown in 71.4% of non-survivors with COVID-19 [35].

In this study, patients with SARS-CoV-2 infection developed a weak innate immune-cell ability to produce cytokines, such as IFN-γ, and showed decreased CD4^+^ and CD8^+^ T cells. SARS-CoV infection increases cytokine expression (e.g., IFN-γ, IL-1, IL-6, IL-10, IL-12, and IL-16) dramatically, and T lymphocytes and their CD4^+^ and CD8^+^ T cells subsets are decreased after the onset of infection [36]. In contrast, HIV-infected patients showed increased mean absolute CD3^+^ T-cell numbers and absolute CD8^+^ T-cell numbers [37]. These data indicate that the inflammatory features of SARS-CoV-2 infection are similar but weaker than that of SARS-CoV infection.

There are limitations to this study. First, this retrospective study mainly analyzed the data related to T cell subset, B cell and NK cell counts, and the function of these cells, while the role of other immune and inflammatory cells infiltrating the pulmonary interstitium remains to be determined. Second, the sex- and age-specific groups had a relatively small number of patients; therefore, these data should be interpreted with caution, and statistical non-significance might not rule out differences among different age-groups. Third, sex and age disparities in COVID-19 cases could be partly explained by differences in sex- and age-related comorbidities; however, the present study did not identify these differences.

In conclusion, SARS-CoV-2 infection mainly induced 1) beneficial sex (female)-related differences regarding reduced COVID-19 disease severity and negative associations with inflammatory responses and liver damage, and 2) harmful age-related differences regarding negative associations with CD8^+^ T cell numbers and positive associations with inflammatory responses and liver damage. Apparently, sex and age are biological variables that should be considered in the prevention and treatment of COVID-19. Hopefully, the simultaneous evaluation of sex and age disparities in COVID-19 may help clinicians provide timely and specific therapy.

## Methods

### Ethics statement

This study conformed to the ethical guidelines of the 1975 Declaration of Helsinki. Ethics approval has been issued by the Ethics Committee of Wenzhou Medical University (Ref 2020002). Given the urgency of the COVID-19 pandemic and global health concerns, the informed consent forms from patients (including the 17-year-old subjects) were waived by the Ethics Committee of Wenzhou Medical University.

### Study design and participants

A total of 693 consecutive patients diagnosed with COVID-19 admitted to 10 hospitals in Wenzhou City, Zhejiang Province, China, from January 17, 2020, to March 20, 2020, were enrolled in this study. Of these patients, only 12 were under 17 years old; thus, 681 met the inclusion criteria for the present analysis (S1 Table). The diagnosis of COVID-19 was performed according to the World Health Organization (WHO) interim guidance and was confirmed by RNA detection of SARS-CoV-2 by hospital clinical laboratories, as described previously [13]. The sixth edition of the COVID-19 diagnosis and treatment plan issued by the National Health Commission was applied to identify and classify COVID-19 patients. Accordingly, COVID-19 patients were classified into four main groups: mild cases (mild symptoms only without radiographic features), moderate cases (fever, respiratory tract symptoms, and pneumonia on chest CT scan), severe cases (respiratory distress syndrome, respiratory rates ≥ 30/min, finger oxygen saturation measured after 5 minutes of rest ≤ 93%, or PaO2 [the arterial oxygen partial pressure]/FiO2 [the inspired oxygen fraction] ≤ 300 mmHg), and critically ill cases (respiratory failure requiring intubation, shock, other organ failures, or admission to the ICU).

To identify the differentiated sex- and age-dependent clinical and immunological features of COVID-19, age-sex bins were categorical variables of the combination of males (n = 362) or females (n = 319) with the following age groups: young, 17–49 years; old, 50–93 years. The sex-specific effects of COVID-19 may be shown by differences between the sexes observed in young patients with relatively high levels of sex hormone and reproductive capability, while the age-specific effects of COVID-19 may be shown by the differences between age groups in younger and older cohorts. Since patients with comorbidities commonly progress to critical conditions, COVID-19 patients with comorbidities were further diagnosed by their clinical and laboratory manifestations after initial medical records sorting. Of 681 patients, 243 suffered from COVID-19 with comorbidities. In this study, NCs (young male [n = 160] and female [n = 103] groups with ages 18–49 years old; old male [n = 50] and female [n = 56] groups with ages 51–81 years old) who came for health check-ups between April 26, 2018, to December 10, 2020, and agreed to contribute their de-identified data to medical research were included as the baseline. For NCs, the inclusion criteria were as follows: age >17 years; absence of COVID-19, absence of systemic inflammatory or infectious diseases, and no medications with known influence on immunological factors.

### Data collection and laboratory procedures

From electronic medical records, epidemiological, demographic, clinical, laboratory, treatment, and outcome data were collected using a standardized data collection form, a slightly modified version of the WHO/International Severe Acute Respiratory and Emerging Infection Consortium case record form for severe acute respiratory infections. Three physicians (CC, BY, and TL) checked all data, and another researcher (SJ) adjudicated any differences in interpretation among the three primary reviewers.

Ethylenediaminetetraacetic acid-anticoagulated peripheral blood samples were collected from patients with COVID-19 tests on the same day before hospitalization. Routine blood examinations included complete blood count (white blood cell, neutrophil, lymphocyte, and platelet counts), serum biochemical tests (including renal and liver function, creatine kinase, and lactate dehydrogenase), myocardial enzymes, CRP, IL-6, IL-2, IL-4, IL-5, IL-10, TNF-α, and IFN-γ counts. Additionally, the levels of the coagulation markers, FIB and d-dimer, were determined. The increase or decrease in cell counts and biochemical parameters were defined as above or under the limit of normal ranges according to local hospital criteria.

### Flow cytometric analysis

To detect the phenotypic characteristics of lymphocytes (CD4^+^ and CD8^+^ T-cells, B-cells, and NK cells), ethylenediaminetetraacetic acid-anticoagulated peripheral blood samples (2 mL) were collected from patients with COVID-19 before initial treatment, and a second sample was collected after 12 days of treatment. Measurements were performed as previously described [26]. Briefly, CD4^+^ and CD8^+^ T-cell, CD19^+^ B-cell, and CD16^+^ CD56^+^ NK-cell staining were performed using the following antibodies: peridinin chlorophyll protein (PerCP)-conjugated anti-human CD3 mAb (BD Biosciences, California, USA), allophycocyanin (APC)-conjugated anti-human CD4 mAb (BD Biosciences), APC/Cy7-conjugated anti-human CD8 mAb (Biolegend, USA), APC-conjugated anti-human CD19 mAb (BD Biosciences), APC-conjugated anti-human CD16, and Brilliant Violet 510 (BV-510)-conjugated anti-human CD56 mAb (Biolegend). The gating strategy of CD4^+^ T-cells, CD8^+^ T-cells, B-cells, and NK cells was executed as CD3^+^CD4^+^, CD3^+^CD8^+^, CD3^−^CD19^+^, and CD3^−^CD16^+^/CD56^+^, respectively, and the cells were analyzed by multiple-color flow cytometry on a BD FACS Canto II flow cytometry system (BD Biosciences).

### Statistical analysis

The results shown are medians (IQR) or numbers (percentages), where appropriate, according to the data distribution. Distributions were compared using D’Agostino & Pearson omnibus normality tests. Considering a statistical adjustment that considers the baseline differences between males and females, we took two different approaches. First, we added four new normal control groups (young male and female groups aged 18–49 years; old male and female groups aged 51–81 years) as the baseline to determine the differences among the age- and sex-matched subjects with or without COVID-19. Second, to deal with parameters that have not been measured in normal controls, we used normal clinical values of men and women from our hospital and compared the patient groups to these backgrounds. Accordingly, the cases above or below the normal limits in men and women were calculated and formed the categorical data. Parametric data with a normal distribution (e.g., hemoglobin) were analyzed using a two-way analysis of variance (ANOVA), followed by Bonferroni’s multiple-comparisons test. If the data did not fulfill the assumptions of parametric statistics, the skewed quantitative data (e.g., counts of white blood cells, neutrophils, lymphocytes, B cells, T cells, NK cells, and CD4^+^ and CD8^+^ T cells, and levels of CRP, IL-6, d-dimer, FIB, ALT, AST, total bilirubin, direct bilirubin, albumin, globulin, hematocrit, creatinine, urea nitrogen, BNP and cTn, and oxygen index) were analyzed using a two-way non-parametric ANOVA (Scheirer-Ray-Hare test), followed by a non-parametric Kruskal–Wallis multiple comparisons test. Categorical variables are shown as frequency (%), and Chi-square tests or Fisher exact tests (e.g., sex and age) and Kruskal–Wallis multiple comparisons tests (e.g., severity and clinical symptoms) were used for comparisons. Spearman correlation coefficients were performed to determine the relationships between age and several variables (neutrophil, lymphocyte, or CD8^+^ T cell counts). MLRA was conducted to determine the associations of cell count (neutrophil, total lymphocyte, B cell, NK cell, CD4^+,^ and CD8^+^ T cell) or CRP, FIB, ALT, and AST levels with sex (male or female), age, and comorbidities (hypertension, diabetes, and both hypertension and diabetes; present or absent). *p* < 0.05 was considered significantly different. Data were evaluated using the SPSS software (SPSS standard, version 25.0; SPSS, Inc., Chicago, IL, USA.).

## Supporting information

S1 Table Sex- and Age-specific Clinical and Laboratory Characteristics of COVID-19 Patients on Admission. (DOCX)

S2 Table Supporting data for Fig 1A. (XLSX)

S3 Table Supporting data for Fig 1B. (XLSX)

S4 Table Supporting data for Fig 1C. (XLSX)

S5 Table Supporting data for Fig 1D. (XLSX)

S6 Table Supporting data for Fig 1E. (XLSX)
