# Supplementary material for: Sex- and age-specific clinical and immunological features of coronavirus disease 2019
Source: PLoS Pathog. 2021 Mar 26;17(3):e1009420. doi: 10.1371/journal.ppat.1009420 (PMC8026060; doi:10.1371/journal.ppat.1009420)
Supplement: S1 Table — (DOCX) [file ppat.1009420.s001.docx]

**S1 Table. Sex- and Age-specific Clinical and Laboratory Characteristics of COVID-19** **Patients on Admission**

|  | **Normal** [**range**](C:/Users/X1/AppData/Local/youdao/dict/Application/8.9.3.0/resultui/html/index.html#/javascript:;)**s** | **All patients (n=681)** | | | **Young (n=374)** | | | **Old (n=307)** | | |
| --- | --- | --- | --- | --- | --- | --- | --- | --- | --- | --- |
|  |  | **Males (n=362)** | **Females (n=319)** | ***P*** | **Males (n=202)** | **Females (n=172)** | ***P*** | **Males (n=160)** | **Females (n=147)** | ***P*** |
| Age, years |  | 47.5(39.0-55.0) | 48.0 (37.5-56.0) | 0.565 | 40.0 (32.0-44.0) | 38.5 (33.0-45.0) | 0.985 | 57.0 (53.0-64.3) | 57.0 (53.0-65.5) | 0.600 |
| Gender |  | 362/681 (53.2%) | 319/681 (46.8%) | 0.020 | 202/374 (54.0%) | 172/374 (46.0%) | 0.028 | 160/307 (52.1%) | 147/307 (47.9%) | 0.294 |
| Comorbidities |  | 139/362 (38.4%) | 104/319 (32.6%) | 0.115 | 56/202 (27.7%) | 33/172 (19.2%) | 0.053 | 83/160 (51.9%) | 71/147 (48.3%) | 0.531 |
| **Severity** | | | | | | | | | | |
| Mild |  | 17/362 (4.7%) | 11/319 (3.4%) | 0.413 | 11/202 (5.4%) | 12/172 (7.0%) | 0.539 | 7/160 (4.4%) | 1/147 (0.7%) | 0.095 |
| Moderate |  | 299/362 (82.6%) | 272/319 (85.3%) | 0.0053 | 169/202 (83.7%) | 153/172 (89.0%) | 0.141 | 129/160 (80.6%) | 117/147 (79.6%) | 0.821 |
| Severe |  | 32/362 (8.8%) | 28/319 (8.8%) | 0.980 | 20/202 (9.9%) | 6/172 (3.5%) | 0.015 | 12/160 (7.5%) | 22/147 (15.0%) | 0.037 |
| Critical ill |  | 14/362 (3.9%) | 8/319 (2.5%) | 0.317 | 2/202 (1.0%) | 1/172 (0.6%) | >0.9999 | 12/160 (7.5%) | 7/147 (4.8%) | 0.320 |
| **Clinical symptoms** | | | | | | | | | | |
| Fever |  | 252/362 (69.6%) | 210/319 (65.8%) | 0.292 | 138/202 (68.3%) | 110/172 (64.0%) | 0.374 | 114/160 (71.3%) | 100/147 (68.0%) | 0.126 |
| Dry cough |  | 153/362 (42.3%) | 100/319 (31.3%) | 0.0033 | 90/202 (44.6%) | 58/172 (33.7%) | 0.033 | 63/160 (39.4%) | 42/147 (28.6%) | 0.046 |
| Sputum production |  | 194/362 (53.6%) | 160/319 (50.2%) | 0.371 | 103/202 (51.0%) | 77/172 (44.8%) | 0.230 | 91/160 (56.9%) | 83/147 (56.5%) | 0.942 |
| Fatigue |  | 109/362 (30.1%) | 99/319 (31.0%) | 0.794 | 55/202 (27.2%) | 49/172 (28.5%) | 0.786 | 54/160 (33.8%) | 50/147 (34.0%) | 0.961 |
| Myalgia or arthralgia |  | 27/362 (7.5%) | 22/319 (6.9%) | 0.700 | 12/202 (5.9%) | 12/172 (7.0%) | 0.684 | 15/160 (9.4%) | 10/147 (6.8%) | 0.410 |
| Headache |  | 33/362 (9.1%) | 37/319 (11.6%) | 0.287 | 18/202 (8.9%) | 19/172 (11.0%) | 0.491 | 15/160 (9.4%) | 18/147 (12.2%) | 0.417 |
| Nausea or vomiting |  | 24/362 (6.6%) | 35/319 (11.0%) | 0.044 | 18/202 (8.9%) | 14/172 (8.1%) | 0.790 | 6/160 (3.8%) | 21/147 (14.3%) | 0.0011 |
| Coeliodynia |  | 8/362 (2.2%) | 16/319 (5.0%) | 0.048 | 4/202 (2.0%) | 7/172 (4.1%) | 0.376 | 4/160 (2.5%) | 9/147 (6.1%) | 0.197 |
| Diarrhea |  | 52/362 (14.4%) | 57/319 (17.9%) | 0.213 | 36/202 (17.8%) | 32/172 (18.6%) | 0.845 | 16/160 (10.0%) | 25/147 (17.0%) | 0.071 |
| Oxygen index, mmHg | >300 | 403.5 (343.1-461.9) | 420.0 (352.6-489.5) | 0.054 | 410.5 (367.1-463.3) | 448.6 (381.3-500.0) | 0.0022 | 394.3 (329.9-459.5) | 392.1 (326.3- 462.3) | 0.965 |
| <300 |  | 39/290 (13.4%) | 30/252 (11.9%) | 0.591 | 20/159 (12.6%) | 6/126 (4.8%) | 0.023 | 19/131 (14.5%) | 24/126 (19.0%) | 0.329 |
| **The clinical routine blood tests** | | | | | | | | | | |
| Leukocyte count, ×10^9^ /L | 4-10 | 5.0 (4.1-6.5) | 4.6 (3.8-5.8) | <0.0001 | 5.0 (3.8-6.4) | 4.3 (3.6-5.4) | 0.0004 | 5.3 (4.4-6.8) | 4.8 (4.0-6.2) | 0.042 |
| >10 |  | 23/357 (6.4%) | 10/316 (3.2%) | 0.049 | 10/199 (5.0%) | 0/171 (0) | 0.0022 | 13/158 (8.2%) | 10/145 (6.9%) | 0.662 |
| Neutrophil count, ×10^9^ /L | 1.8-6.3 | 3.2 (2.3-4.4) | 2.9 (2.1-3.8) | 0.0007 | 3.0 (2.2-4.1) | 2.7 (1.8-3.6) | 0.0023 | 3.4 (2.6-5.1) | 3.1 (2.3-4.4) | 0.066 |
| <1.8 |  | 43/355 (12.1%) | 54/315 (17.1%) | 0.065 | 28/198 (14.1%) | 39/171 (22.8%) | 0.031 | 15/157 (9.6%) | 15/144 (10.4%) | 0.803 |
| >6.3 |  | 38/355 (10.7%) | 17/315 (5.4%) | 0.013 | 14/198 (7.1%) | 1/171 (0.6%) | 0.004 | 24/157 (15.3%) | 16/144 (11.1%) | 0.286 |
| Lymphocyte count, ×10^9^ /L | 1.1–3.2 | 1.2 (0.9-1.6) | 1.3 (0.9-1.5) | 0.501 | 1.3 (1.0-1.7) | 1.3 (1.0-1.6) | 0.870 | 1.1 (0.7-1.4) | 1.1 (0.9-1.5) | 0.233 |
| NLR |  | 2.6 (1.7-4.1) | 2.2 (1.6-3.4) | 0.0036 | 2.3 (1.5-3.3) | 2 (1.5-2.9) | 0.024 | 2.9 (2.0-5.0) | 2.7 (1.8-3.9) | 0.054 |
| Hematocrit, % | Male:40–50  Female:35–45 | 42.3% (39.1-45.0) | 37.5% (35.3-39.8) | <0.0001 | 43.3% (41.2-45.7) | 38.2% (35.4-40.2) | <0.0001 | 40.7 % (37.4-43.4) | 37.1% (35.1-39.7) | <0.0001 |
| <35% |  | 25/349 (7.2%) | 71/313 (22.7%) | <0.0001 | 7/195 (3.6%) | 36/170 (21.2%) | <0.0001 | 18/154 (11.7%) | 35/143 (24.5%) | 0.004 |
| Hemoglobin, g/L |  | 145.0（134.3-154.0） | 126.0（119.0-134.0） | <0.0001 | 149.5（140.0-156.0） | 128.0（122.0-135.0） | <0.0001 | 139.0（128.0-151.0） | 124.0（116.0-131.5） | <0.0001 |
| **Coagulatory and fibrinolytic indicators** | | | | | | | | | | |
| Fibrinogen, g/L | 2-4 | 4.1 (3.3-5.1) | 3.7 (2.9-4.5) | <0.0001 | 3.9 (3.1-4.9) | 3.3 (2.7-4.3) | <0.0001 | 4.4 (3.5-5.5) | 4.0 (3.3-4.8) | 0.025 |
| >4 |  | 168/326 (51.5%) | 113/283 (39.9%) | 0.039 | 81/181 (44.8%) | 44/148 (29.7%) | 0.0052 | 87/145 (60.0%) | 69/135 (51.1%) | 0.135 |
| D-dimer, mg/L | <500 | 270.0 (170.0-500.0) | 280.0 (160.0-440.0) | 0.547 | 220.0 (149.3-387.5) | 240.0 (134.0-350.0) | 0.883 | 350.0 (215.0-635.0) | 320.0 (195.0-600.0) | 0.438 |
| >500 |  | 83/317 (26.2%) | 60/275 (21.8%) | 0.216 | 33/174 (19.0%) | 20/141 (14.2%) | 0.259 | 50/143 (35.0%) | 40/134 (29.9%) | 0.364 |
| **Indicators of organ damages** | | | | | | | | | | |
| cTn, pg/ml | <10 | 12.0 (9.0-20.0) | 10.0 (3.0-14.0) | 0.0041 | 12.0 (5.0-20.0) | 10.0 (1.0-12.0) | 0.0005 | 12.0 (10.0-20.0) | 12.0 (10.0-20.0) | 0.518 |
| >10 |  | 143/255 (56.1%) | 107/223 (48.0%) | 0.0771 | 73/134 (54.5%) | 47/115 (40.9%) | 0.0322 | 70/121 (57.9%) | 60/108 (55.6%) | 0.7263 |
| BNP, pg/ml | <100 | 47.5 (10.0-100.0) | 42.0 (10.0-100.0) | 0.702 | 25.0 (10.0-100.0) | 28.5 (10.0-100.0) | 0.456 | 53.0 (11.1-100.0) | 88.0 (18.1-103.0) | 0.250 |
| Albumin (A), g/L | 40-55 | 40.2 (37.0-43.4) | 39.9 (37.3-42.6) | 0.759 | 41.8 (38.8-44.6) | 41.3 (39.0-43.1) | 0.349 | 38.5 (35.4-41.3) | 38.9 (35.8-41.4) | 0.541 |
| Globulin (G), g/L | 20-30 | 28.6 (25.4-31.9) | 28.9 (26.1-31.9) | 0.441 | 27.6 (24.8-31.0) | 28.6 (25.4-3 0.8) | 0.350 | 29.6 (26.6-33.4) | 29.3 (26.6-33.3) | 0.911 |
| A/G | 1.5-2.5:1 | 1.4 (1.2-1.6) | 1.4 (1.2-1.6) | 0.553 | 1.5 (1.3-1.7) | 1.5 (1.3-1.6) | 0.204 | 1.3 (1.1-1.5) | 1.3 (1.1-1.5) | 0.542 |
| ALT, IU/L | Male:5-40  Female:5-35 | 29.0 (20.0-44.0) | 17.0 (12.0-25.0) | <0.0001 | 27.5 (18.0-43.8) | 15.0 (11.0-22.0) | <0.0001 | 29.0 (20.0-45.0) | 20.0 (14.0-29.5) | <0.0001 |
| Male>40, female >35 |  | 102/353 (28.9%) | 39/313 (12.5%) | <0.0001 | 57/199 (28.6%) | 14/169 (8.3%) | <0.0001 | 45/154 (29.2%) | 25/144 (17.4%) | 0.016 |
| AST, IU/L | 8-40 | 26.0 (21.0-37.0) | 22.0 (18.0-29.0) | <0.0001 | 25.0 (20.0-32.3) | 20.0 (17.0-23.0) | <0.0001 | 30.0 (22.7-46.4) | 26.0 (19.0-33.3) | 0.0047 |
| >40 |  | 72/319 (22.6%) | 30/293 (10.2%) | <0.0001 | 28/180 (15.6%) | 7/157 (4.5%) | P<0.001 | 44/139 (31.7%) | 23/136 (16.9%) | 0.0044 |
| Total bilirubin, µmol/L | 3.4-17.1 | 11.6 (8.7-15.6) | 9.5 (6.3-14.3) | <0.0001 | 11.7 (9.0-15.9) | 9.0 (6.2-13.8) | <0.0001 | 11.6 (8.4-15.3) | 10.1 (6.7-15.0) | 0.012 |
| Direct bilirubin, µmol/L | 0-3.4 | 4.2 (3.0-6.0) | 3.5 (2.7-5.2) | <0.0001 | 4.4 (3.2-6.0) | 3.3 (2.5-4.9) | <0.0001 | 4.0 (3.0-6.0) | 3.8 (2.7-5.5) | 0.100 |
| Urea nitrogen, mmol/L | Male:2.3-7.1  Female:1.8-6.1 | 4.1 (3.5-5.0) | 3.4 (2.8-4.2) | <0.0001 | 3.9 (3.4-4.5) | 3.1 (2.7-3.6) | <0.0001 | 4.5 (3.8-5.6) | 4.0 (3.1-4.9) | <0.0001 |
| Male>7.1, Female >6.1 |  | 22/354 (6.2%) | 16/313 (5.1%) | 0.5397 | 5/199 (2.5%) | 2/169 (1.2%) | 0.5842 | 17/155 (11.0%) | 14/144 (9.7%) | 0.7241 |
| Creatinine, µmol/L | Male:62-115  Female:53-97 | 75.0 (66.6-88.0) | 57.9 (52.0-65.0) | <0.0001 | 77.0 (67.1-88.0) | 56.0 (50.9-62.0) | <0.0001 | 74.0 (65.0-85.0) | 60.0 (53.0-68.0) | <0.0001 |
| Male>115, Female >97 |  | 9/353 (2.5%) | 6/314 (1.9%) | 0.579 | 3/198 (1.5%) | 1/170 (0.6%) | 0.627 | 6/155 (3.9%) | 5/144 (3.5%) | 0.855 |
| **Inflammatory factors, cytokines and interleukins** | | | | | | | | | | |
| CRP, mg/L | <10 | 12.5 (5.0-36.6) | 6.6 (2.9-20.4) | <0.0001 | 10.0 (4.8-25.9) | 5.0 (2.0-13.0) | <0.0001 | 15.6 (5.1-43.1) | 13.0 (5.0-28.9) | 0.060 |
| >10 |  | 199/353 (56.4%) | 135/314 (43.0%) | 0.0006 | 98/196 (50.0%) | 52/169 (30.8%) | 0.0002 | 101/157 (64.3%) | 83/145 (57.2%) | 0.207 |
| IFN-γ, pg/ml |  | 2.4 (1.4-3.3) | 2.5 (1.5-3.4) | 0.578 | 2.5 (1.5-3.3) | 2.5 (1.7-3.5) | 0.342 | 2.3 (1.3-3.0) | 2.1 (1.3-3.2) | 0.885 |
| TNF-α, pg/ml |  | 1.1 (0.6-2.5) | 1.0 (0.7-1.9) | 0.379 | 1.3 (1.0-2.5) | 1.0 (0.9-1.9) | 0.425 | 1.0 (0.6-2.5) | 1.0 (0.5-2.0) | 0.741 |
| IL-2, pg/ml |  | 1.5 (1.0-2.5) | 1.5 (1.2-2.5) | 0.437 | 1.5 (1.1-2.5) | 1.7 (1.2-2.5) | 0.625 | 1.5 (0.9-2.5) | 1.5 (1.2-2.4) | 0.394 |
| IL-4, pg/ml |  | 1.7 (1.1-2.6) | 1.7 (1.2-2.4) | 0.491 | 1.9 (1.3-2.9) | 1.8 (1.1-2.5) | 0.478 | 1.6 (1.0-2.5) | 1.6 (1.3-2.2) | 0.942 |
| IL-5, pg/ml |  | 1.5 (1.1-2.0) | 1.2 (1.0-1.5) | 0.047 | 1.5 (1.3-1.8) | 1.2 (1.0-1.9) | 0.322 | 1.4 (1.0-2.2) | 1.2 (1.0-1.4) | 0.242 |
| IL-6, pg/ml |  | 5.5 (3.3-16.5) | 4.0 (2.4-11.3) | 0.010 | 4.1 (3.0-13.8) | 3.7 (2.1-6.5) | 0.022 | 6.8 (3.7-29.5) | 4.4 (2.6-15.8) | 0.065 |
| IL-10, pg/ml |  | 3.5 (2.0-4.9) | 3.1 (0.9-4.4) | 0.037 | 3.3 (1.6-4.4) | 2.5 (1.0-3.8) | 0.043 | 3.6 (2.4-5.8) | 3.5 (0.9-5.0) | 0.247 |
| **Lymphocyte and subsets** | | | | | | | | | | |
| Lymphocytes /μl | 1100–3200 | 1200 (900-1600) | 1262 (938-1513) | 0.501 | 1290 (985-1700) | 1300 (1000-1569) | 0.870 | 1100 (700-1400) | 1100 (900-1500) | 0.233 |
| <1100 |  | 138/356 (38.8%) | 114/316 (36.1%) | 0.473 | 63/199 (31.7%) | 50/171 (29.2%) | 0.615 | 75/157 (47.8%) | 64/145 (44.1%) | 0.459 |
| CD4^+^T cells /μl |  | 421.0 (279.0-609.0) | 508.0 (327.5-650.8) | 0.077 | 436.0 (328.0-590.0) | 521.0 (366.0-588.0) | 0.266 | 379.5 (233.8-614.5) | 483.0 (299.5-663.0) | 0.128 |
| CD8^+^ T cells /μl |  | 282.0 (178.0-405.0) | 291.5 (177.3-429.3) | 0.820 | 330.0 (234.0-500.0) | 357.0 (271.0-443.0) | 0.642 | 233.5 (138.5-328.5) | 226.0 (141.0-364.0) | 0.614 |
| CD4^+^T /CD8^+^T cell ratio | 1.5-2.0 | 1.6 (1.1-2.2) | 1.7 (1.2-2.3) | 0.210 | 1.4 (1.0-1.8) | 1.3 (1.1-1.8) | 0.468 | 1.9 (1.4-2.6) | 1.9 (1.5-2.8) | 0.483 |
| T cells /μl |  | 708.0 (497.0-1032.0) | 824.7 (535.8-1062.3) | 0.225 | 767.0 (567.0-1183.0) | 901.0 (617.0-1088.0) | 0.419 | 597.5 (444.8-918.8) | 758.0 (483.5-1055.5) | 0.222 |
| B cells /μl |  | 151.5 (94.8-222.3) | 159.0 (115.0-233.0) | 0.500 | 146.0 (103.0-232.0) | 128.0 (91.0-169.0) | 0.219 | 155.0 (93.0-207.0) | 177.0 (121.3-258.3) | 0.084 |
| NK cells /μl |  | 212.0 (118.0-336.0) | 195.5 (133.0-276.0) | 0.520 | 222.5 (154.0-330.8) | 194.0 (132.6-300.0) | 0.203 | 184.0 (89.5-338.0) | 203.0 (139.0-271.0) | 0.725 |

Data are median (IQR), n/N (%). p values were calculated by Kruskal-Wallis test, χ² test, Yates’ continuity corrected chi-square test or Fisher’s exact

test, as appropriate. ALT=alanine aminotransferase.cTn=cardiac troponin. BNP=Brain natriuretic peptide. ALT=alanine aminotransferase. AST=aspartate aminotransferase. CRP=c-reactive protein. IL=interleukin. IFN-γ=interferon-γ.
